# Modified Navigation Instructions for Spatial Navigation Assistance Systems Lead to Incidental Spatial Learning

**DOI:** 10.3389/fpsyg.2017.00193

**Published:** 2017-02-13

**Authors:** Klaus Gramann, Paul Hoepner, Katja Karrer-Gauss

**Affiliations:** ^1^Biological Psychology and Neuroergonomics, Berlin Institute of TechnologyBerlin, Germany; ^2^Center for Advanced Neurological Engineering, University of California, San Diego, San DiegoCA, USA; ^3^Human Machine Systems, Berlin Institute of TechnologyBerlin, Germany

**Keywords:** navigation assistance, spatial memory, automation, cognitive decline

## Abstract

Spatial cognitive skills deteriorate with the increasing use of automated GPS navigation and a general decrease in the ability to orient in space might have further impact on independence, autonomy, and quality of life. In the present study we investigate whether modified navigation instructions support incidental spatial knowledge acquisition. A virtual driving environment was used to examine the impact of modified navigation instructions on spatial learning while using a GPS navigation assistance system. Participants navigated through a simulated urban and suburban environment, using navigation support to reach their destination. Driving performance as well as spatial learning was thereby assessed. Three navigation instruction conditions were tested: (i) a control group that was provided with classical navigation instructions at decision points, and two other groups that received navigation instructions at decision points including either (ii) additional irrelevant information about landmarks or (iii) additional personally relevant information (i.e., individual preferences regarding food, hobbies, etc.), associated with landmarks. Driving performance revealed no differences between navigation instructions. Significant improvements were observed in both modified navigation instruction conditions on three different measures of spatial learning and memory: subsequent navigation of the initial route without navigation assistance, landmark recognition, and sketch map drawing. Future navigation assistance systems could incorporate modified instructions to promote incidental spatial learning and to foster more general spatial cognitive abilities. Such systems might extend mobility across the lifespan.

## Introduction

The increasing use of in-car navigation systems, primarily due to the apparent advantages such as improved wayfinding, driving performance, and safety (Gelau and Krems, 2004; Blanco et al., 2006; Lee and Cheng, 2008; Liang and Lee, 2010) changes the way drivers interact with their environment and how they apply spatial cognitive skills. Following simple turn-by-turn navigation instructions reduces the requirements necessary to focus on aspects of the environment that are relevant to the navigation task. Such systems thus free up cognitive resources that would otherwise be needed to plan movement in space allowing a focus on other tasks unrelated to navigation (see e.g., Münzer et al., 2006). North-up map displays allow for better spatial knowledge acquisition while track-up map systems can be used to support immediate decision-making during navigation (Rizzardo et al., 2013). By providing step-by-step instructions, however, track-up map displays support wayfinding accuracy at the expense of spatial knowledge acquisition leading to spatial cognitive deskilling (Aporta et al., 2005; Burnett and Lee, 2005; Münzer et al., 2012).

Several navigation assistance systems have been developed to overcome spatial deskilling and to improve spatial learning, which can be described as a sequential development of spatial knowledge starting with the representation of single landmarks and progressing through route knowledge to survey knowledge (Siegel and White, 1975). Landmarks are external reference points that can serve as key navigation cues which are easily remembered and recognized. Several empirical studies have proved the benefits of landmarks in navigation instructions (Streeter and Vitello, 1986; Wochinger and Boehm-Davis, 1997; Burnett, 2000; May and Ross, 2006). Their visibility, familiarity, uniqueness, permanence, and the usefulness of their location impact their effective use for navigation (Carr and Schissler, 1969; Allen et al., 1978; Green et al., 1995; Burnett et al., 2001). Based on landmark knowledge and through repetitive travel between two landmarks, route knowledge develops in a second step of spatial learning. Route knowledge allows for automated travel between two well-known landmarks while routes can be indicated on map displays to support navigational learning (e.g., Burnett and Lee, 2005). The final level of spatial knowledge is configural knowledge, comprising landmarks and their interconnection and allowing for computing shortcuts that have never been navigated before (Siegel and White, 1975). While the sequential approach to spatial learning can be argued (Ishikawa and Montello, 2006; Gramann, 2013), the different levels of spatial knowledge provide a useful framework to evaluate spatial learning during automated navigation.

Navigation assistance systems that aim at fostering spatial learning can be placed in four different categories described along the dimensions of spatial knowledge (familiar vs. unfamiliar environment) and the amount of effort involved in spatial learning (low vs. high learning effort).

One navigation system aiming to increase spatial learning is the scaffolded navigation support (see e.g., Patel et al., 2006; Fox and Boyer, 2013). This approach can be used in partially known environments and leads to higher learning effort (high familiarity/high effort) by reducing the granularity of navigational instructions as the user’s experience with the environment increases. A corresponding system integrates existing environmental knowledge and supports users only as much as necessary, thus increasing user effort as information has to be actively retrieved from memory. Burnett and Lee (2005) developed a system that trains spatial skills in familiar areas without increasing the spatial learning effort (high familiarity/low effort) during the navigation task by displaying previously taken routes. This approach is supposed to enable users to integrate the currently driven route into an existing representation of other routes in the form of a cognitive map.

Approaches that foster spatial learning in unfamiliar areas are associated with additional learning effort for the driver by forcing her/him to become involved with the environment. By providing, for example, only an aerial perspective of the environment (allocentric reference frame) instead of a first-person perspective (egocentric reference frame), configural knowledge can be acquired. However, this requires the driver to mentally transform the spatial information perceived from a first-person perspective (egocentric, e.g., turning to the right) and to align this with the provided map reference frame (allocentric, e.g., turning west; see e.g., Münzer et al., 2012). An alternative restriction of navigation assistance would let the driver decide *when* to receive navigational instructions or would ask the driver questions about her/his immediate environment while driving (Parush et al., 2007). While all approaches with high spatial learning effort aim at supporting spatial learning, they decrease the amount of resources available for safe vehicle control.

Systems with low learning effort used in environments with low or high familiarity demonstrated an improvement in spatial learning by integrating a compass into the navigation map telling the driver the cardinal directions and supporting the correct integration of the driven route into an allocentric cognitive map (Oliver and Burnett, 2008; but see Waters and Winter, 2011). Leshed et al. (2008) proposed the use of landmarks as reference points within the visualization of the navigational map, or to connect otherwise rather indistinct navigational information to real objects within the driver’s environment.

In summary, navigation assistance systems that allow spatial learning in familiar and unfamiliar environments would be the most desirable. Like any other navigation assistance, a learning-oriented system has to secure vehicle safety and fulfill the primary purpose to effectively guide navigation without compromising behavior in the primary driving task.

In the present study we test incidental spatial learning with a learning-oriented navigation system that uses different navigation instructions and compare these to standard navigation instructions. Modified navigation instructions carry information describing the kind of and affordance, i.e., any possible action on a landmark at decision-relevant points. These modified instructions could be presented with or without personal-relevant information regarding individual preferences (taste in food, music, favorite animals, etc.). As an example, the affordance of the landmark restaurant would be to eat a meal or the personally favorite dish. We predicted that the use of the three navigation assistance systems would lead to comparable experienced effort of the users, and none of the navigation assistance systems were expected to have an impact on driving behavior. The levels of processing theory put forward by Craik and Lockhart (1972) and the extension by Jacoby and Craik (1979), served as framework to describe the levels of processing of spatial information in the environment. We assumed differences in processing of spatial information from simple perception of attributes of the surrounding (e.g., color and form of buildings) to deeper levels of processing including processing of the identity (e.g., residential building) and potential function of environmental features (e.g., friends’ apartment). The former would reflect superficial processing according to Craik and Lockhart (1972) while the latter two would reflect increasingly deeper processing of features of the environment. By providing navigation instructions that highlight a specific building at a navigation relevant intersection, we aimed at creating landmarks. Additionally, providing contrasting information should foster deeper processing of the spatial feature “landmark” that could then be associated with a navigation decision (e.g., turn right). A deeper level of processing of spatial features should lead to an integration of the same within an existing memory network. Consequently, when the driver receives standard navigation instructions (i.e., no highlighting of spatial features) the surroundings of navigation relevant intersections should be superficially processed and no spatial knowledge acquisition should be observed. In contrast, intersections receiving contrast modified information to highlight specific spatial features of the surroundings (i.e., landmarks) were expected to be processed on a deeper level and spatial memory performance should improve compared to the standard instructions. As the standard instructions provided no contrasting information for spatial features, we did not systematically vary levels of processing but tested the general improvement of deeper processing of contrasted spatial features versus generic navigation instructions. Finally, based on the “self-reference-effect” discovered by Rogers et al. (1977) which describes a deeper processing of information that is tied to personal concepts such as one’s own name or personal interests, highlighting a specific landmark in combination with personal-relevant information was expected to lead to the best spatial knowledge acquisition.

## Materials and Methods

### Participants

A total of 58 participants were invited to participate in the study. To control for the impact of age (Cornoldi and Vecchi, 2004) and gender on spatial navigation (e.g., Lawton, 1994; Sandstrom et al., 1998), experimental groups were homogenized, resulting in 20 participants in the standard condition and 19 in both modifier conditions. Out of the initial 58 participants, eight participants discontinued the experiment due to simulator sickness, and another four were excluded due to problems understanding the instructions. The final sample comprised 19 men and 27 women in three groups with 17 participants in the standard condition, 16 in the contrast modifier, and 13 in the personal-reference modifier condition. All participants were licensed drivers aged 22 to 38 (mean = 26.5, *SD* = 3.54) with at least 2 years’ driving experience (mean = 7.87, *SD* = 3.38). Most participants drove less than 10,000 km a year (84.78%) while only a few drove between 10,000 and 20,000 km (8.69%) or more than 20,000 km (6.53%). This research complied with the tenets of the Declaration of Helsinki and was approved by the Institutional Review Board. All participants gave their informed consent to participate in the study, which was compensated for with course credits or a financial reimbursement.

### Experimental Conditions

Three different navigation instructions were investigated in a between-subjects design with a control group using a standard instruction and two experimental groups using modified navigation instructions. The standard instructions at decision points represented the “classic” navigation system with simple turn instructions (“Please turn right at the next intersection”). The modified navigation instructions provided additional information, referred to as ‘modifiers,’ related to landmarks encountered at decision points. Landmarks were salient objects like buildings or other stationary structures that could be used for spatial orienting. Landmarks could be relevant or irrelevant with respect to the driving task. A relevant landmark indicated a turn instruction while irrelevant landmarks were located along the route and required no change in direction.

The first navigation instruction modification included contrast modifiers, i.e., contrasting specific landmarks with the surrounding environment by providing explicit information regarding the identity and affordance of the landmark. To this end, the reference to the generic landmark “intersection” in the control condition was replaced by information regarding the identity of a specific landmark (e.g., “the bookstore”) and a short sentence describing the landmark’s affordance (e.g., “Here you can buy books.”). An example of a contrast-modified condition would be: “Please turn right at the concert hall. Here you can attend concerts.” The second instruction modification included personal-relevant modifiers. In addition to increasing the saliency of landmarks by referring to the identity of the landmark (e.g., “the bookstore”), this group received personal information on a landmark at a decision point (“Here you can buy your favorite book, Moby Dick.”). Favorite activities – hobbies, books, movies, etc. – were collected in a questionnaire before the experiment. The following example describes a navigation instruction that includes a personal movie preference related to the landmark “movie theater”: “Please turn right in front of the movie theater. There you can watch your favorite movie, Zoolander.” Personal-relevant navigation instructions were adapted to each participant. To this end, a text-to-speech system was integrated (Google TTS API) that read out the relevant text from an XML file and generated an audio file.

**FIGURE 1:**
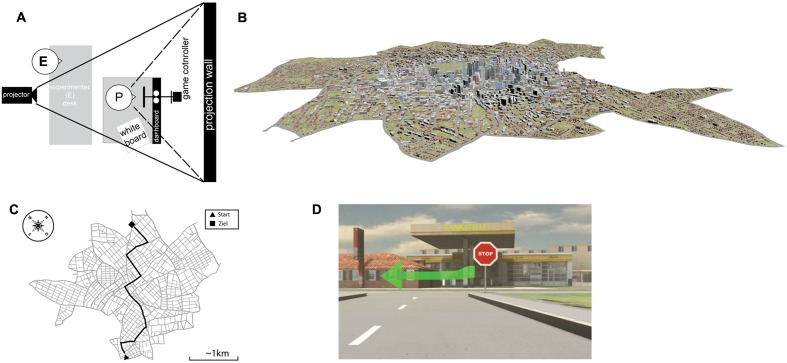
**(A)** Schematic of the experimental setup using a projector behind the experimenter (E) located behind the participant (P). Participants were seated in a mock-up of a VW Touran with the steering wheel connected to a game controller. To their right, a whiteboard was located to allow questionnaire answers and map drawing. **(B)** Full rendering of the environment used with suburban areas and the city center as displayed in **(C)** as a real map. **(D)** Example of a left-turn instruction at a landmark (gas station).

Participants were seated in a driving simulator consisting of a seat box and a projector displaying the scene approximately 1.9 m in front of the participants (viewing angle of ∼90° horizontally and ∼54° vertically, dependent on the exact position and height of the participant; see **Figure 1A**). All groups navigated through the same suburban and urban environment to reach a predefined destination (**Figures 1B,C**), receiving navigation instructions at identical intersections that required them to turn left or right. All groups received the same visual information at decision points in the form of a hologram-based navigation instruction on a simulated head-up display (**Figure 1D**). In addition, auditory navigation instructions were provided that differed between experimental conditions.

### Measurements and Apparatus

#### Driving Simulator

The driving simulator mimicked a VW Touran interior and integrated the Game Controller “MOMO Racing Force Feedback Wheel” to assess the steering behavior and pedal actuation. Due to the limited maximum angle of rotation of the controller of around 110° the sensitivity of the control was adapted and the sensitivity was reduced with increasing speed by a gain factor of 0.1.

#### Virtual Environment and Route

A virtual city model was created with City Engine^1^ allowing free travel through a total area of approximately 36 km^2^ with a road network of approximately 20 km^2^, consisting of suburban areas and a city center. After creating a homogeneous city with global landmarks such as the skyline, local landmarks (e.g., sports stadium) were positioned along the route. These landmarks stood out from the environment with respect to their function and appearance compared to other buildings. For example, the gas station (**Figure 1D**) had a significant architecture including signs that deviated from the buildings in the area and that thus increased the contrast with neighboring buildings.

Two landmark types were used: (1) relevant landmarks that indicated a navigation decision (turn left/right) were located at decision points, unpredictably positioned on the left or right of the street, irrespective of the turning instruction; and (2) irrelevant landmarks that were located along the route between two decision points and that did not require any turn maneuver. Irrelevant landmarks were used to control for general spatial learning effects and were not combined with navigation instructions.

The complexity of the route was set to seven decision points and avoided predictable patterns in the route (e.g., not alternating systematically between left and right). The open source simulator software, OpenDS^2^, was adjusted to the requirements of the experiment including modifications to save the driving behavior of participants, such as turn signals, steering wheel position, and pedal positions. In addition, the vehicle position was continuously recorded to identify navigation errors. Both auditory and visual navigation instructions were triggered at predefined positions along the route.

### Procedure

After arriving at the lab, participants were asked to provide personal information, such as favorite items and activities that was later used for the navigation instruction. Participants were introduced to the driving simulator and then got acquainted with driving in the setup in a 5-min training session. Subsequently, participants navigated the virtual city in one of the three navigation instruction conditions (standard, contrast modifier, personal-relevant modifier). They were instructed to follow the navigation instructions and to adhere to speed limits and general traffic regulations.

This first navigation phase took 8 min on average and was followed by a first NASA Task Load Index questionnaire (NASA-TLX; Hart and Staveland, 1988). Participants were then presented with a landmark recognition task in which 19 pictures of landmarks were presented in random order. Seven pictures contained relevant landmarks that were previously presented together with a navigation instruction (turn left/right). Relevant landmarks were presented from the same perspective that participants encountered with presentation of the navigation instruction when approaching the intersection. In the recognition task the landmarks were displayed without any navigation instructions or the turn arrow. In addition, six pictures containing irrelevant landmarks that were not associated with a navigation instruction were included, presented at the same perspective that participants encountered when approaching them. Finally, six pictures containing novel landmarks were included that were not encountered during navigation. Pictures were shown on the screen while participants remained in the car and responded by either turning the wheel to indicate the turn direction that was associated with the landmark in the initial navigation phase or by applying the accelerator, or the brakes, to indicate landmarks during straight sections or unknown landmarks, respectively.

Subsequently, a second navigation phase was conducted in which participants were instructed to drive the same route again without navigation assistance. In case of navigation errors where the distance to the correct route surpassed 15 m (e.g., after an incorrect turn) the participant was stopped and placed at the last correct point on the route. After the second navigation phase participants filled out the second NASA-TLX questionnaire and answered additional questions regarding their gaming experience, driving behaviors, and use of navigation assistance systems. In a final test, participants were asked to draw a map of the environment. Overall, the procedure took 50 min on average.

### Dependent Measures

To assess the subjective workload during the driving task, participants completed the unweighted NASA-TLX. Performance-based measures were derived from the participants’ driving behavior, under the assumption that driving performance degrades with an increasing workload, usually reflected in parameters of lateral and longitudinal vehicle control (Angell et al., 2006). To this end, deviation from the ideal line and the number of direction changes was calculated. In addition, deviations from the indicated speed were analyzed as well as the number of changes in the accelerator pedal position indicating participants’ effort to comply with the speed requirements.

Spatial knowledge acquisition was measured by the number of correctly recognized landmarks in the landmark recognition task. Because a correct response required an association of a landmark with a navigation decision (turn left, turn right, accelerate, or brake), the landmark recognition task required landmark as well as route knowledge. Simple landmark knowledge was assessed by quantifying the naming of a landmark (vs. the absence of a landmark) in the sketch map, irrespective of the position of the landmark. Because the classical navigation instruction provided no landmark names, any concept that reflected the landmarks identity (e.g., writing down “animals” or drawing a “giraffe” for the landmark zoo) was counted as correct.

In addition to the number of correctly named landmarks in the sketch maps, the number of correct route segments indicating connections between successive landmarks and the correct association of landmark and navigation decision (turn left, right, or straight ahead) was extracted from the sketch map, providing additional information on participants’ route knowledge. For the number of correct route segments, the correct placement of pairs of landmarks was assessed as the correct spatial order of two landmarks in sequence connected by a route segment. In case one landmark was missing the next pair of landmarks had to be in the correct sequence to be counted as correct. In case a landmark was placed incorrectly, the landmark was dismissed and the next landmark was used as reference to score the relation to the following landmark. The correct placement of landmarks was again measuring participants’ route knowledge as it required knowledge of the relative sequence of two landmarks. As a final measure for route knowledge, navigation decisions were counted as correct when the routes that connected successive landmarks included a turn in the right direction for relevant landmarks. The relative placement of different landmarks regarding the principle directions of the route in the environment (e.g., from south to north and west to east) was not considered for this measure.

Due to the generally low quality of the sketch maps the current study did not quantify measures reflecting survey knowledge. Because the naming of landmarks had an impact on how many route segments could be correct, the different route knowledge measures extracted from the sketch map were partially dependent.

## Results

### Mental Workload

#### NASA-TLX

The mental demand subscale of the NASA-TLX was analyzed using a mixed measures design with the three different navigation assistance conditions (standard, contrast modifier, and personal-relevant modifier) as a between-subjects measure and the first and second navigation phase as repeated measures. In case of significant effects Tukey’s honestly significant difference (Tukey-HSD) tests were computed for *post hoc* comparisons.

**FIGURE 2:**
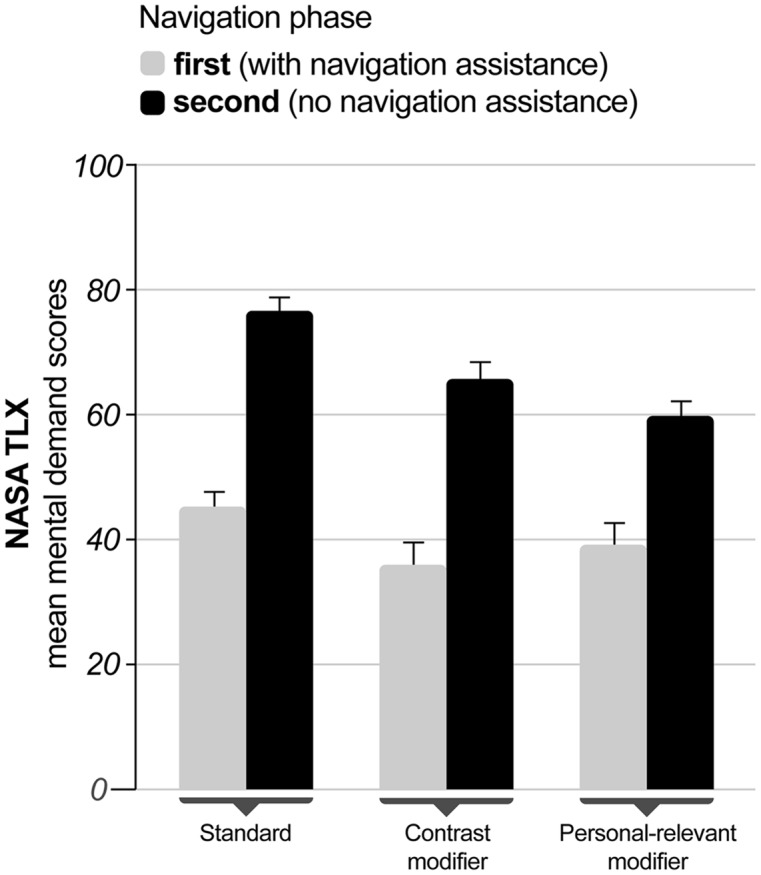
**NASA Task Load Index (NASA-TLX) scores for the mental demand subscale as a function of navigation instruction for the first (with instruction, light gray bars) and second (without instruction, black bars) navigation phase.** Error bars indicate one standard error.

The analysis revealed a main effect of navigation phase [*F*_(1,43)_ = 53.01, *p* < 0.001; η = 0.552] with mean mental demand ratings of 40.06 and 67.26 for the first and second navigation phase, respectively (see **Figure 2**). There was a trend toward significance for the instruction conditions [*F*_(2,43)_ = 3.09, *p* = 0.056; η^2^ = 0.126] but no interaction of both factors (*p* > 0.485). Tukey-HSD comparisons revealed none of the contrasts to be significant (all *p*s > 0.083).

### Driving Parameters

#### Lane and Speed-Keeping

To investigate lane and speed-keeping performance, separate ANCOVAs were computed for (i) deviations from the ideal line, and (ii) the number of direction changes (reflecting lane keeping), as well as (iii) deviations from the speed limit, and (iv) changes in the accelerator pedal (reflecting speed-keeping). The navigation assistance condition (standard, contrast modifier, and personal-relevant modifier) was entered as a between-subjects factor and the navigation phase (with vs. without navigation assistance) was computed as repeated measures. The NASA-TLX mental demand subscales for the first and second navigation phase were entered as covariates.

The results demonstrated no main effect or interaction of the covariates with any other factor (all *p*s > 0.22). The deviation from the ideal line [*F*_(1,41)_ = 0.326, *p* = 0.571; η^2^ = 0.008] and the number of direction changes [*F*_(1,41)_ = 0.108, *p* = 0.305; η^2^ = 0.026] was comparable in both navigation phases. There was no impact of instruction condition on the deviation from the ideal line [*F*_(2,41)_ = 0.869, *p* = 0.427; η^2^ = 0.041] or the number of direction changes [*F*_(2,41)_ = 0.150, *p* = 0.861; η^2^ = 0.007] and none of the interaction terms reached significance (*p*s > 0.842) indicating that lane-keeping behavior was comparable for both navigation phases when mental demand was accounted for. With only slightly higher deviations from the speed limit during the first (mean = 11.38 km/h, *SD* = 1.37) as compared to the second (mean = 10.97 km/h, *SD* = 1.38) navigation phase neither the instruction conditions [*F*_(2,43)_ = 0.22, *p* = 0.80; η^2^ = 0.010] nor the navigation phase [*F*_(2,41)_ = 0.429, *p* = 0.65; η^2^ = 0.021] were significant. The interaction term also failed to demonstrate an impact on speed deviations [*F*_(2,43)_ = 0.36, *p* = 0.697; η^2^ = 0.017]. The number of changes by the accelerator pedal was comparable for the first and the second navigation phase [*F*_(1,41)_ = 0.324, *p* = 0.57; η^2^ = 0.008] and neither the instruction conditions [*F*_(2,43)_ = 0.24, *p* = 0.976; η^2^ = 0.001] nor the interaction of both factors reached significance [*F*_(2,43)_ = 0.51, *p* = 0.609; η^2^ = 0.023].

### Spatial Learning

#### Landmark Recognition

Landmark recognition performance was analyzed using ANCOVA with navigation assistance conditions (standard, contrast modifier, and personal-relevant modifier) as a between-subjects measure and landmark type (relevant, irrelevant, and novel) as a repeated measure entering the subscale mental demand of the NASA-TLX after the first navigation phase as a covariate.

**FIGURE 3:**
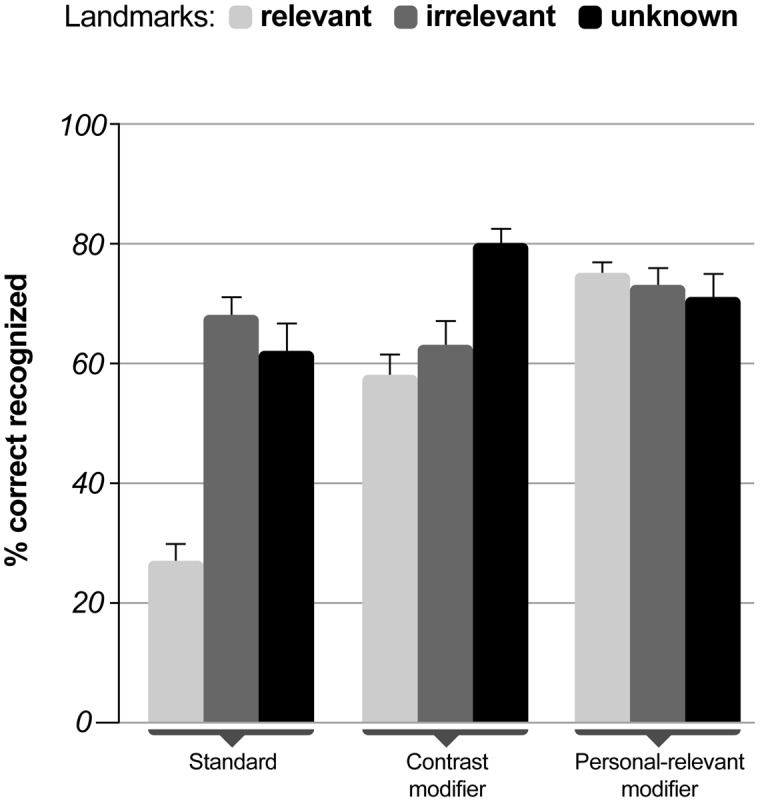
**Mean percent correct recognized landmarks as a function of navigation instruction dependent on landmark category (relevant, irrelevant, and novel).** The rate of correctly recognized landmarks was defined as the ratio of correctly recognized landmarks and the number of all landmarks in the respective landmark category (relevant, irrelevant, and novel) with a value of one indicating 100% correct responses. Error bars indicate one standard error.

There was no effect of the mental demand on landmark recognition [*F*_(1,42)_ = 1.09, *p* < 0.303; η^2^ = 0.025]. The landmark type showed no impact on recognition [*F*_(2,84)_ = 1.23, *p* < 0.299; η^2^ = 0.028] while the instruction condition [*F*_(2,42)_ = 8.66, *p* < 0.001; η^2^ = 0.292] and the interaction of landmark type and instruction condition reached significance [*F*_(4,84)_ = 5.49, *p* = 0.001; η^2^ = 0.207] after controlling for the effect of mental demand. **Figure 3** displays the mean correct recognition rates for the different navigation instructions as a function of the landmark type.

Pairwise comparisons for the main effect of instruction conditions using Sidak correction for multiple comparisons revealed that the standard instructions differed significantly from both the contrast modified (*p* = 0.018) and the personal-relevant modified (*p* = 0.001) navigation instructions. The modified navigation instructions did not differ with respect to the number of correctly recognized landmarks (*p* = 0.540). Bonferroni corrected comparisons revealed that the recognition rates for relevant landmarks in the standard instruction condition were significantly lower than for all other landmark types in all conditions (all *p*s > 0.012) while no differences in the recognition rates were observed for relevant landmarks in the contrast modified and personal-relevant compared to any other instruction condition (all *p*s > 0.17).

#### Navigation Errors in the Second Navigation Phase

An ANCOVA with the navigation assistance conditions (standard, contrast modifier, and personal-relevant modifier) as a between-subjects measure and the NASA-TLX subscale mental demand for the second navigation phase as a covariate revealed no impact of mental demand on the number of navigation errors [*F*_(1,42)_ = 2.036, *p* < 0.161; η^2^ = 0.046]. The main effect of the navigation instruction [*F*_(2,42)_ = 15.148, *p* < 0.001; η^2^ = 0.419] was significant, showing more errors for participants receiving standard navigation instructions during the first navigation phase (*M* = 4.94, *SD* = 2.36) as compared to contrast modifiers (*M* = 2.25, SD = 1.91) or personal-relevant modifiers (*M* = 1.54, *SD* = 0.88). Pairwise comparisons with Sidak corrections showed significant differences between the standard and both the contrast modified (*p* < 0.001) and the personal-relevant instructions (*p* < 0.001) while the latter two conditions did not differ (*p* = 0.229).

#### Correct Route Segments in Sketch Maps

**FIGURE 4:**
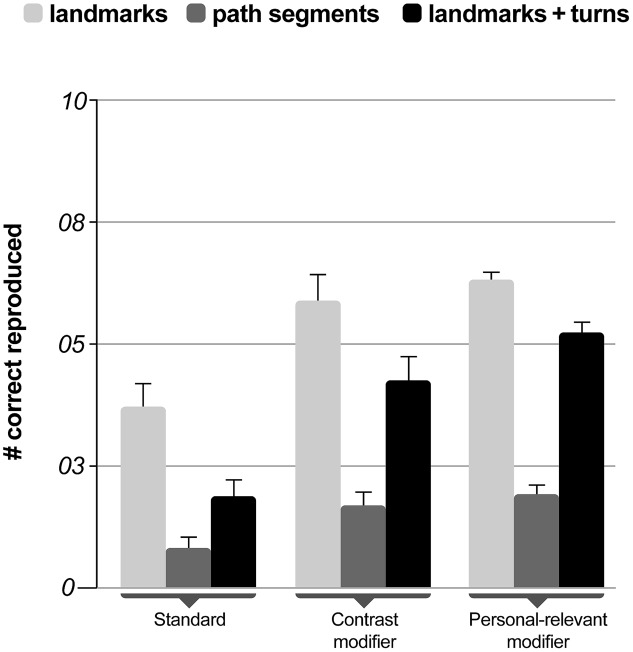
**Mean percent correct reproduced features in the sketch map task as a function of navigation instruction.** Error bars indicate one standard error.

The mean number of correctly placed landmarks (relevant and irrelevant), the number of correct path segments, and the combination of correctly placed landmarks including navigation decisions as a classification of reproduced sketch map features were the lowest in the standard navigation instruction condition (*landmarks*: 3.71, path: 0.82, decision: 1.88) and increased for navigation instructions with contrast modifiers (*landmarks*: 5.88, path: 1.69, decision: 4.25) to navigation instructions with personally relevant information (*landmarks*: 6.31, path: 1.92, decision: 5.23; **Figure 4**). These three dependent measures derived from participants’ sketch maps were entered as dependent measures in separate ANCOVAs with navigation assistance systems (standard, contrast modifier, and personal-relevant modifier) as a between-subjects factor and the NASA-TLX subscale mental demand of the second navigation phase as a covariate.

The analyses revealed that the main effect of the instruction condition was significant for the number of correctly placed landmarks [*F*_(2,42)_ = 3.27, *p* = 0.048; η^2^ = 0.135]. Navigation instructions did not impact the number of correctly reproduced path segments in the sketch map [*F*_(2,42)_ = 2.37, *p* = 0.106; η^2^ = 0.101] but revealed an effect on the correctly reproduced combination of landmarks and turns [*F*_(2,42)_ = 7.28, *p* = 0.002; η^2^ = 0.257]. None of the analyses showed a significant effect of mental demand (all *p*s > 0.431) and Sidak corrected multiple comparisons of the main effects revealed significant differences only for the combination of landmarks and turns between standard instructions and contrast modified (*p* = 0.021) and personal-relevant instructions (*p* = 0.002)n without difference between the latter two conditions (*p* = 0.586).

## Discussion

In this study, we investigated whether modified navigation instructions lead to incidental spatial learning of the environment. Three different navigation assistance systems were compared: a system that used standard instructions indicating the turn direction, and two systems using modified instructions aimed at creating a contrast for landmarks located at decision-relevant points to support an efficient and deeper cognitive elaboration of the information and, as a consequence, to learn spatial aspects of the environment.

Comparing the two navigation phases with and without navigation assistance revealed increased subjective workload ratings for the phase without navigation instructions. In combination with changes in the driving performance from the first to the second navigation phase, this result supports the assumption that the simulated driving task without support was more demanding for participants. Importantly, the subjectively experienced workload was the same for all navigation instruction conditions, demonstrating that modified instructions did not cause higher mental demand. Comparable lateral and longitudinal vehicle control further indicated that modified navigation instructions had no impact on driving behavior.

The results further confirmed the hypotheses that participants receiving contrasting and personally relevant information incidentally learned the spatial aspects of the environment. There was a significant reduction in the number of navigation errors when the same route had to be navigated without assistance, indicating spatial learning through modified instructions. Spatial learning also became evident in the increased rate of recognized landmarks. In addition, the analysis of map drawings revealed more correctly remembered landmarks and combination of landmarks and navigation decisions in the modified navigation instructions as compared to the standard navigation instruction. While the results from the sketch map analyses support the other measures of spatial learning it should be noted that only one rater analyzed the map drawings without being blind to the participants’ experimental condition. In summary, these results demonstrate incidental spatial knowledge acquisition for landmark and route knowledge (e.g., Stern and Leiser, 1988).

Contrary to our hypothesis, spatial knowledge acquisition was comparable for contrast and personal relevant instructions. The absence of specific instruction effects might have been due to insufficient power based on the relatively low number of participants and potential differences in spatial abilities between participants (Gramann et al., 2005, 2010; Ishikawa and Montello, 2006; Goeke et al., 2015). While in the landmark recognition task the highest recognition rates were observed for landmarks with personal-relevant modifiers, the differences to contrast modified landmarks did not reach significance. Due to the design of the present study, it would be difficult to differentiate whether improved landmark recognition was based on a personal reference effect or the depth of processing of this additional information. Contrast modifiers did not provide specific information (i.e., the explanation “you can buy books” in a bookstore might be considered redundant). Thus, improved landmark recognition in the personal-relevant modifier condition could be based on either a deeper level of processing or the self-reference effect.

Also contrary to our expectations, recognition rates for relevant landmarks were somewhat lower in the standard condition compared to irrelevant or unknown landmarks. This result stands in marked contrast to studies demonstrating higher parahippocampal activity related to navigationally relevant landmarks (Janzen and van Turennout, 2004) and greater viewpoint-independent encoding of relevant landmarks (Han et al., 2012). One possible explanation could be that the process of making a turn as compared to driving straight ahead demanded more attention from the participants, with fewer resources left to process the surrounding area. However, because landmarks were already visible before the turn it seems more likely that the standard auditory navigation instructions (e.g., “turn left at the intersection”), drew the participant’s attention to the turning indicator and the intersection itself, rather than to the spatial features surrounding the intersection. This might have resulted in an inhibition of landmark learning because the landmarks were simply not processed. The significant difference between the control and the modified instruction conditions could therefore be a mixture of suppressed spatial learning in standard navigation instructions and the promotion of spatial learning in the modified navigation instruction conditions due to an additional auditory coding of meaningful information for landmarks that are made salient. These results are also in line with other studies by Janzen and colleagues when assuming that relevant landmarks were simply not attended to and were less likely encoded as a relevant landmark in the classical instruction condition. Physiological measures could provide deeper insights into these hypotheses and would allow for a comparison with earlier studies demonstrating increased processing of relevant landmarks (Janzen and van Turennout, 2004; Janzen et al., 2008; Han et al., 2012).

It could be seen as a general limitation of the current experimental design that learning the surrounding environmental features could be related to the drivers’ attention being directed toward landmarks in the modified instruction conditions. In this sense, modified instructions differed from standard instructions in two ways: first, they increased the saliency of a landmark using verbal cues, and secondly, they provided additional contrasting information. This kind of dual-coding advisory turn indication was demonstrated to have a significant impact not only on landmark recognition but also on configural knowledge acquisition (Rizzardo and Colle, 2013). As landmarks were not made salient in the standard instruction condition, learning effects might be independent from the semantics of navigation instructions.

While the main concern with the current study design was the short delay between spatial learning and recognition, the results of the present study are very promising. Because the tests were conducted within 1 h it is too early to conclude that the results indicate long-term memory effects which are at the core of our spatial learning approach. Nonetheless, our results provide a first indication that modified turn-by-turn instructions indeed lead to better spatial learning without increasing effort during the navigation task. Future studies will investigate prolonged delays between learning and testing to understand whether incidental spatial long-term learning takes place. The system safety is warranted while spatial learning takes place simultaneously. If such a system, in combination with available information of visited places, information from social networks, browsing activities, and direct input through the user is used on a daily basis, it might well lead to an augmentation of spatial cognitive abilities due to constant incidental learning of spatial information that can easily be integrated into an existing memory network.

### Outlook

A learning-oriented navigation system could be applied in private and professional contexts, as in the training of taxi or bus drivers who have to form spatial representations as part of their job. Learning-oriented, automated navigation should also be considered in the broader context of learning-oriented systems. Incidental lifelong training of spatial cognitive skills might help to augment spatial cognitive abilities in users that interact with such technical systems. The continuous training of spatial cognitive processes might thus trigger neural plasticity that extends cognitive abilities and might improve general memory capacity up to an old age.

## Ethics Statement

This study was carried out in accordance with the recommendations of the German Psychological Association. The protocol was approved by the ethics committee of the Institute for Psychology and Ergonomics of the Berlin Institute of Technology.

## Author Contributions

KG developed the idea and experimental protocol, supervised the experiment, analyzed the data, and wrote the manuscript. PH programmed the VR environment, implemented the protocol, collected, and analyzed the data. KK-G contributed to the data analyses and was involved in writing the manuscript.

## Conflict of Interest Statement

The authors declare that the research was conducted in the absence of any commercial or financial relationships that could be construed as a potential conflict of interest.

## Footnotes

1 http://www.esri.com/software/cityengine

2 http://www.opends.de
